# Innovative Green Chemistry Approach to Synthesis of Sn^2^^+^-Metal Complex and Design of Polymer Composites with Small Optical Band Gaps

**DOI:** 10.3390/molecules27061965

**Published:** 2022-03-18

**Authors:** Shujahadeen B. Aziz, Muaffaq M. Nofal, Mohamad A. Brza, Niyaz M. Sadiq, Elham M. A. Dannoun, Khayal K. Ahmed, Sameerah I. Al-Saeedi, Sarkawt A. Hussen, Ahang M. Hussein

**Affiliations:** 1 Hameed Majid Advanced Polymeric Materials Research Lab., Physics Department, College of Science, University of Sulaimani, Qlyasan Street, Sulaimani 46001, Kurdistan Regional Government, Iraq; niyaz.sadiq@univsul.edu.iq (N.M.S.); khayal.ahmed@univsul.edu.iq (K.K.A.); sarkawt.hussen@univsul.edu.iq (S.A.H.); ahang.hussein@univsul.edu.iq (A.M.H.); 2 Department of Civil Engineering, College of Engineering, Komar University of Science and Technology, Sulaimani 46001, Kurdistan Regional Government, Iraq; 3 Department of Mathematics and Science, Prince Sultan University, P.O. Box 66833, Riyadh 11586, Saudi Arabia; muaffaqnofal69@gmail.com; 4 Medical Physics Department, College of Medicals & Applied Science, Charmo University, Chamchamal, Sulaimania 46023, Iraq; mohamad.brza@gmail.com; 5 Department of Mathematics and Science, Woman Campus, Prince Sultan University, P.O. Box 66833, Riyadh 11586, Saudi Arabia; elhamdannoun1977@gmail.com; 6 Department of Chemistry, College of Science, Princess Nourah bint Abdulrahman University, P.O. Box 84428, Riyadh 11671, Saudi Arabia; sialsaeedi@pnu.edu.sa

**Keywords:** Sn^2+^-PPHs metal complex, UV-Vis, XRD and FTIR analyses, optical property, bandgap analysis

## Abstract

In this work, the green method was used to synthesize Sn^2+^-metal complex by polyphenols (PPHs) of black tea (BT). The formation of Sn^2+^-PPHs metal complex was confirmed through UV-Vis and FTIR methods. The FTIR method shows that BT contains NH and OH functional groups, conjugated double bonds, and PPHs which are important to create the Sn^2+^-metal complexes. The synthesized Sn^2+^-PPHs metal complex was used successfully to decrease the optical energy band gap of PVA polymer. XRD method showed that the amorphous phase increased with increasing the metal complexes. The FTIR and XRD analysis show the complex formation between Sn^2+^-PPHs metal complex and PVA polymer. The enhancement in the optical properties of PVA was evidenced via UV-visible spectroscopy method. When Sn^2+^-PPHs metal complex was loaded to PVA, the refractive index and dielectric constant were improved. In addition, the absorption edge was also decreased to lower photon. The optical energy band gap decreases from 6.4 to 1.8 eV for PVAloaded with 30% (*v*/*v*) Sn^2+^-PPHs metal complex. The variations of dielectric constant versus wavelength of photon are examined to measure localized charge density (*N*/m*) and high frequency dielectric constant. By increasing Sn^2+^-PPHs metal complex, the *N*/m* are improved from 3.65 × 10^55^ to 13.38 × 10^55^ m^−3^ Kg^−1^. The oscillator dispersion energy (*E_d_*) and average oscillator energy (*E_o_*) are measured. The electronic transition natures in composite films are determined based on the Tauc’s method, whereas close examinations of the dielectric loss parameter are also held to measure the energy band gap.

## 1. Introduction

According to a recent study, the optical properties of polymer composites (PCs) have piqued the interest of a lot of academics because of their extensive application in a variety of sectors, including solar cells, optoelectronic device and light-emitting diode (LED) [1,2]. Inorganic particles are commonly found in polymers, which are thought to be an outstanding host material. Studies on the optical characteristics of PVA based on metal complexes have been conducted in the literature [3,4].The insertion of inorganic particles into the host polymer might result in a significant alteration in the host’s characteristics due to their high surface to bulk ratio [5].Green techniques have been widely reported as potential approaches for the synthesis of inorganic particles, with the results being shown to be safe and environmentally benign [6].

Alkaloids, amino acids, catechins, theavins, isomers of theavins, and other elements make polyphenols (PPHs) in black tea (BT). The most obvious molecular or chemical structures of the ingredients of BT have also been described in other investigations [7,8]. Dryan et al. [8] recently published a study which discovered that PPHs components are abundant in the BT aqueous mixture. PPHs conjugate, PPHs, and polymerized phenolic structure are the key elements of BT. In addition, black, green, and white tea all have a unique blend of conjugated flavonoids [9]. In earlier researches, it was found that extract solutions of black and green tea play a key role in lowering the polar polymers optical band gap including PMMA and PVA [10,11]. The extract tea solution contained PPHs, carboxylic acid groups, and hydroxyl group, according to the FTIR study [11]. As a result of the discoveries of experimental studies, the tea extract solution contains a large number of active ligands and functional groups, which are essential for complex formation with polymers and/or transition metal salts.

As a green technique, BT plant extract solutions can be utilized to synthesize Sn^2+^-PPHs metal complexes. These solutions are high in PPHs, which have a significant interaction with the Sn^2+^ ion, forming a Sn^2+^-PPHs metal complex. Zielinski et al. described the primary ingredients and uses of tea leaves, for instance, PPHs and caffeine [12].

Earlier research has shown that functional groups and PPHs in tea extract solutions can capture the cations of heavy metals to crate metal complexes [3,4]. Metal complexes are combined with PVA polymer to create PCs with high-performance optical characteristics in the current work. This process is a new green technique to make PCs with adjustable optical band gaps. Electrical and optical properties of polymers have attracted researchers’ interest in recent years due to their widespread application in optical systems and their superior interference, reflection, anti-reflection, and polarization capabilities [13].In recent studies, it has been discovered that PCs with low band gap energy (*E_g_*) and large absorption play a key role in photonics and optoelectronic device applications [14]. Hasan et al. [15] reported that the use of nanotube-PCs in photonics is due to the composites’ good optical absorptions, which cover a wide spectrum range from UV to near IR [15]. Organic–inorganic hybrid (PCs) serve as an active or passive layer in optoelectronic devices for instance large refractive index films, protective coatings, thin films, LEDs, solar cells, transistors, and waveguide materials play a vital role in various applications [16].

The goal of this research is to create PCs with a low energy bandgap (*E_g_*). Because of the good optical properties, the green method might be used to make PCs with low *E_g_*. The findings of this research can be regarded as a novel PCs approach. The optical dielectric function was accurately used in this study to experimentally detect the different types of optical transition between the conduction band and the valence band. Sn^2+^-polyphenol complex has a strong effect on the decrease of optical band gap in comparison with the other fillers for example nanoparticles (NPs). Aziz et al. [17] prepared PCs based on polystyrene. In their research, copper (Cu) powder was loaded into the polystyrene from 0 to 6 wt.%. Upon the incorporation of 6 wt.% Cu, the *E_g_* decreased from 4.05 to 3.65 eV. Aziz et al. [18], in another work, added copper monosulfide (CuS) NPs into methyl cellulose (MC) polymer to prepare polymer nanocomposites based on MC. The *E_g_* of MC decreased from 6.2 to 2.3 eV by the incorporation of 0.08 M of CuS NPs. In the current work, we observed that the *E_g_* decreased from 6.4 to 1.8 eV for PVA loaded with 30% (*v*/*v*) of Sn^2+^-polyphenol complex. Thus, based on the band gap analysis result, the green method is an appropriate for fabricating PCs with low value of *E_g_*.

## 2. Methodology

### 2.1. Materials

Sigma-Aldrich provided PVA powder (MW ranging from 85,000 to 124,000) and Tin(II) chloride (SnCl_2_) (MW = 189.6 g/mol). The BT leaf was bought from a nearby market.

### 2.2. Sample Preparation

**Scheme 1:**
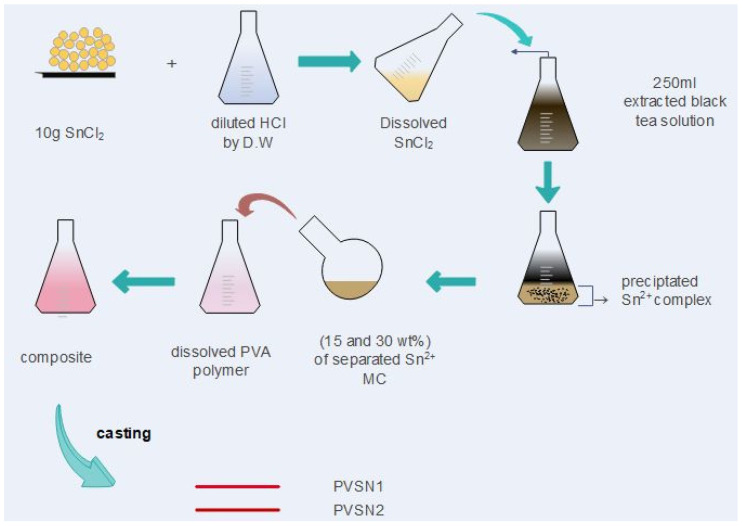
Schematic diagram of sample preparation.

The use of distilled water (D.W.) in the extraction of tea leaves is required. The steps are as follows: In the absence of sunshine, 50 g of BT leaf was placed in 250 mL D.W.at almost 90 °C. The resultant extract solution was filtered by (Whatman paper 41, cat. no. 1441) with a pore radius (20 µm) to thoroughly remove the residues after standing for 10 min. 200 mL HCl was diluted into 400 mL of D.W. and then used it to dissolve 10 g of SnCl_2_ in a separate flask. The Sn^2+^-PPHs metal complex was then made by adding SnCl_2_ solution to the extract tea leaf solution and stirring for 10 min at 80 °C. The complexation between Sn^2+^-metal ions and PPHs was confirmed by the color change of the extract solution from dark to green at the top of the beaker and formation of sediment as clouds at the bottom of the beaker. The complex solution was allowed to cool to room temperature. These complexes were detached in 100 mL of D.W. after numerous washings of the Sn^2+^-PPHs metal complexes with D.W. The solution cast approach was used to produce composite samples made up of PVA loaded with Sn^2+^-PPHs metal complex. To begin, a PVA solution was made by adding 1 g of PVA to 40 mL of D.W., stirring for 1 h at roughly 80 °C, then cooling to room temperature. Different volumes of the complex solution, ranging from 0 to 30% (*v*/*v*), were added to the homogenous PVA solution in 15% (*v*/*v*) increments. The resulting solutions were stirred for approximately 50 min. PVSN0, PVSN1 and PVSN2 were used to represent 0% (*v*/*v*), 15% (*v*/*v*) and 30% (*v*/*v*) of the loaded complex solution, respectively. To cast the manufactured films, the contents of the mixture were poured into petri dishes and allowed to dry at ambient temperature. The samples were dried more using blue silica gel desiccant prior to characterization. Pure PVA and composite films have thicknesses ranging from 0.012 to 0.015 cm. A pictorial sample preparation of PCs consists of Sn^2+^-PPHs metal complex and PVA is shown in Scheme 1.

### 2.3. Measurement Techniques

X-ray diffraction (XRD) patterns were analyzed at room temperature using a Bruker AXS diffractometer (Billerica, MA, USA) with a 40-kV voltage and 45-mA current. The composite films were examined using a Nicolet iS10 FTIR spectrophotometer (Perkin Elmer, Yokohama, Japan) with a resolution of 2 cm^−1^ in the range of 450 and 4000 cm^−1^. A Jasco V-570 UV-vis-NIR spectrophotometer (JASCO, Tokyo, Japan) was used to record the samples UV-vis absorption spectra. For measuring UV-Vis for the liquid samples (Sn^2+^-PPHs complexes), firstly, two cuvettes filled with distilled water were used for correcting background and then one of the cuvettes was removed while another cuvette was left and used as a reference sample. The absorbance of the liquid samples (Sn^2+^-PPHs complexes) was measured in comparison to the reference sample. For measuring FTIR for the liquid samples (Sn^2+^-PPHs complex), Sn^2+^-PPHs complexes were coated on the standard glass slides and then dried at room temperature until evaporated. The dried Sn^2+^-PPHs complexes were scratched on the glass slides to create powders form. Then potassium bromide (KBr) (100 mg) was added to the Sn^2+^-PPHs (1 mg) powders and then the powders were combined in a mortar and finally turned to pellets in a sample holder.

When Sn^2+^-PPHs complexes were added to the dissolved PVA, PVA composite films were created. For measuring UV-Vis and FTIR for the solid composite films, firstly the UV-Vis spectroscopy and FTIR devices with air and without any samples were corrected for background and then the UV-Vis and FTIR spectra were measure for the composite films.

## 3. Results and Discussion

### 3.1. UV-Vis and FTIR Study of Sn^2+^-PPHs Metal Complex

It’s worth noting that coordination chemistry involves complicated coordinated systems, complex molecules, or simply complexes. The lights and empty orbital metallic core that are coordinated by donors of electron pairs are examples of coordination compounds [19]. Coordination chemistry produces metal complexes that have a significant absorption characteristic in visible areas.

**Figure 1:**
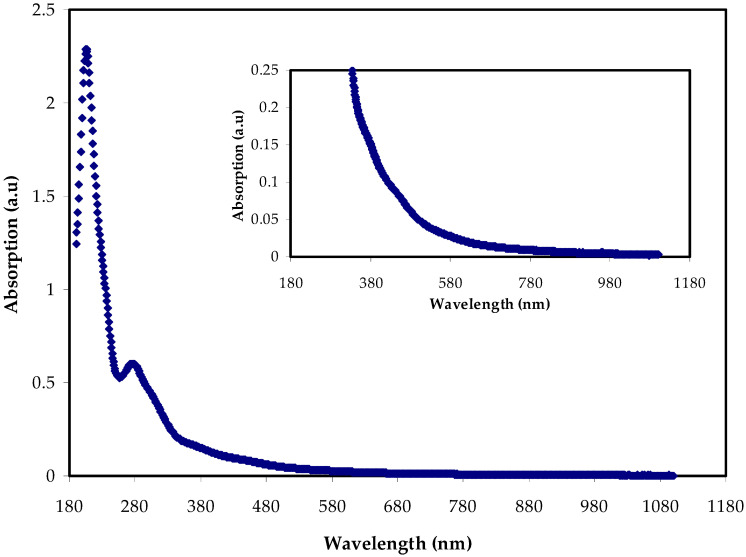
UV-visabsorption spectrum for Sn^2+^-PPHs metal complex.

Figure 1 shows the absorption spectrum of the complicated colloidal suspension (Sn^2+^-PPHs metal complex), which is equivalent to that of organometallic-based materials and semiconductors [20]. The absorption spectrum is notable for covering the whole visible range. The Sn^2+^-complex displays absorption even at high wavelength ranges to near-infrared, as shown in the inset of Figure 1, meaning that increase the optical absorption and light harvesting. Such absorption of a broader spectrum of solar radiation shows the use of such material for various applications. The current UV-Vis data for metal-PPHs complexes generated using green methods are similar to those shown by other studies [21].

The transition of electron of n−π* of methylxanthines, catechins, and caffeine emerges as an absorbance band between 200 and 350 nm. The C=O chromophore in caffeine has a band absorbance of 278 nm [22,23]. Surface plasmon resonance (SPR) absorption band in UV-visible range is required for metallic’s with diameters in the nano range [24]. Nevertheless, the lack of this band in the current Sn^2+^-PPHs complex suggests that the PPHs capping inhibited the complex system’s metal properties from forming on particle surfaces. Cu NPs in chitosan-based PEs produce an SPR band in the region of 500 and 800 nm, according to earlier study [25].

### 3.2. FTIR Studyof BTand Sn^2+^-PPHs Metal Complex

**Figure 2:**
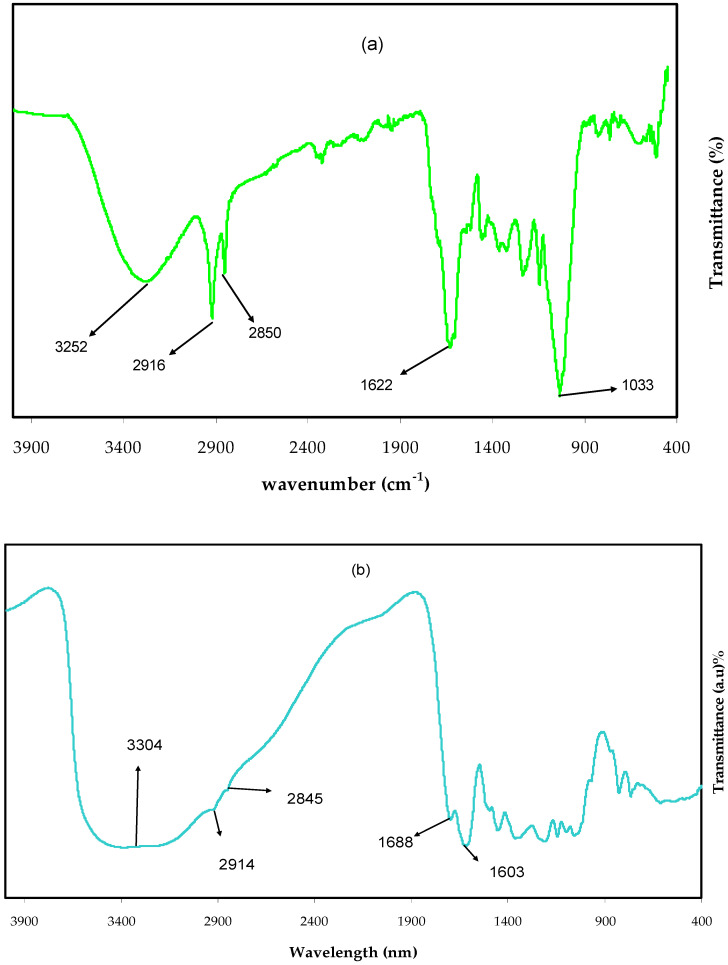
Spectra of the FTIR for the (**a**) extracted BTleaf and (**b**) colloidal Sn^2+^-PPHs metal complex.

The FTIR spectra of the extracted BT’s are shown in Figure 2a. The emergence of many peaks is viewed as the FTIR spectrum’s main characteristic. The C-H stretching of carboxylic acid and aliphatic group is responsible for the current peaks in the range of 2913–2847 cm^−1^ [26].The existence of a band at 1623 cm^−1^ could also be used to identify the aromatic ring’s C=C stretch [16,19,26]. It is worth mentioning that the existing FTIR spectrum’s overall characteristics match those found in prior investigations [27,28]. The caffeine spectrum has recently been discovered to feature several changes in the range between 1700 and 400 cm^−1^ (Figure 2a). The existence of a range of functional groups with stretching and binding movements, for instance carbonyl, methyl, imidazole, and pyrimidine fragments, can be seen in these changes [29]. The key functional groups in tea, as shown by the FTIR spectra, are PPHs, carboxylic acid, and amino acids. PPHs have been demonstrated to interact with the metal cation to produce colloidal metal-PPHs complex solutions, according to the literature [30].

Figure 2b shows the FTIR spectrum of the Sn^2+^-PPHs metal compound. A sequence of peaks in the region between 1700 and 400 cm^−1^ can be noticed in both Figure 2a,b; their intensities were nearly modified as common characteristics.

FTIR is used to investigate the colloidal Sn^2+^-PPHs metal as one of the characteristics of the Sn^2+^-complex. Wang et al. [21] investigated the use of eucalyptus leaf extract in the production of Fe-PPHs complexes. The interaction between Fe^2+^ and PPHs was stressed as the mechanism for forming the complex.

In the FTIR spectrum of Sn^2+^-PPHs metal complexes, the distinctive bands of BT are repeated, but the peak intensities have reduced (see Figure 2b).

**Figure 3:**
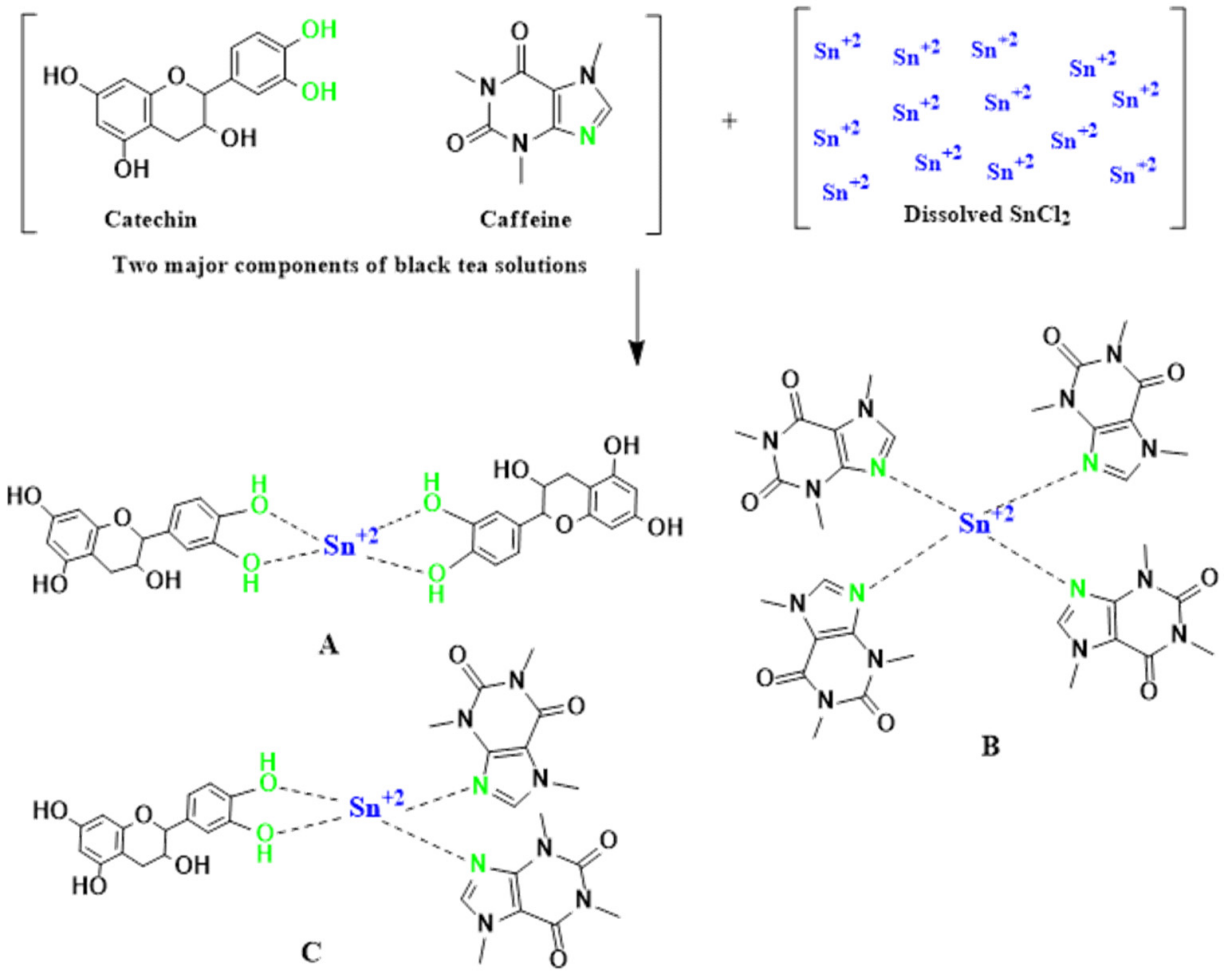
The proposed chemical structure for the Sn^2+^-PPHs metal complex formation mechanism. (**A**) Sn^2+^-catechin metal complex, (**B**) Sn^2+^-caffeine complex, and (**C**) interaction between Sn^2+^ and both caffeine and catechin.

When the Sn^2+^-PPHs metal complexes are formed, the bands of 2914 and 2850 cm^−1^ in BT bands have changed, and currently come into view at 2914 and 2845 cm^−1^, respectively. This is described on the basis of the generation of coordination interactions between PPHs and Sn^2+^ ions, which results in vibrational decrease and arises in reducing mass. More specifically, the development of coordination bonds among PPHs and Sn^2+^ metal ions are caused by an attraction between the Sn^2+^ ion’s empty orbitals and the ligand pairs [31]. In the following section, the mechanism of coordination between the Sn^2+^ ion and the interested ligands is schematically described, as shown in Figure 3. Wang et al. [21] used a range of extracts, containing melaleucanesophila, eucalyptus tereticornis, and rosemarinus, to synthesize and characterize iron-PPHs complexes. The authors have demonstrated that iron ions and PPHs interact together, forming iron-PPHs complexes. FTIR was used by Coinceanainn et al. to investigate the complexation between the aflavinand aluminum (III). The polyphenolic chemicals are ligands observed in BT extract [32].

FTIR analysis can be used to inspect the interaction nature between Sn^2+^ metal cations and caffeine, as well as PPHs in tea extracts, as illustrated in Figure 3. The interaction of the Sn^2+^-metal ion with BT extract includes the production of a number of complexes (see Figure 3). Metal ion interactions with tea components have already been confirmed [26,33]. Figure 3 depicts three different potential complexes. Sn^2+^-PPHs metal complex, as expected (see Figure 3A), and Sn^2+^-caffeine are also expected (Figure 3B). Additionally, as demonstrated in Figure 3C, there is a probability of interaction between Sn^2+^ and both caffeine and PPHs in a complex. The EPR technique had previously been used to study the development of complexes by PPHs in BT extract and metal ions [33]. The nature of metal complex production was investigated utilizing FTIR in the current work.

**Figure 4:**
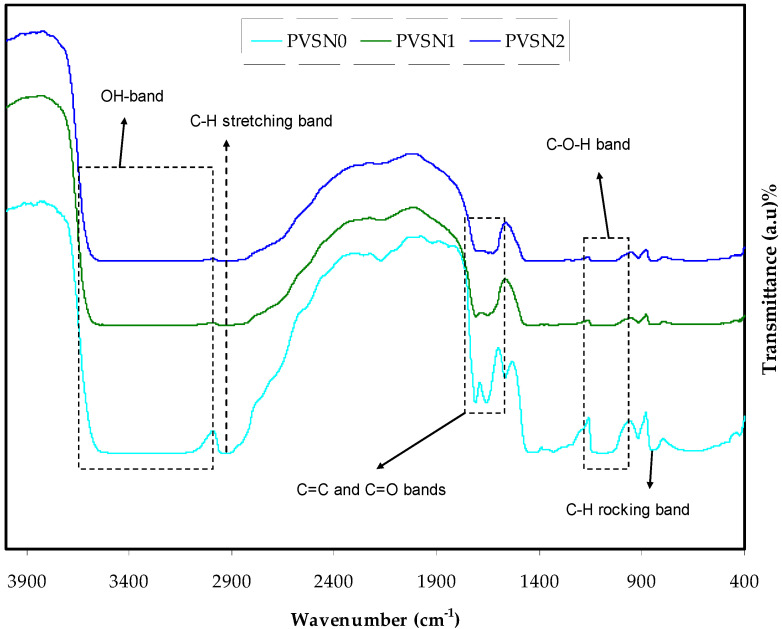
FTIR spectra for pure PVA and loaded films.

The use of FTIR spectroscopy to measure interactions among ions or atoms in a PE or PC systems is crucial. The interactions that occur can cause a shift in the polymer electrolyte’s vibrational modes [34]. Figure 4 shows the FTIR of PVA and PVA loaded samples, respectively. C–H rocking of PVA is attributed to the band at 821 cm^−1^ [34]. For samples containing 15% (*v*/*v*) and 30% (*v*/*v*) of dopant material, this peak changes to 818 and 838 cm^−1^, respectively.

Pure PVA absorption maxima at 1313 and 1410 cm^−1^ have been ascribed to C–OH plane bending and CH_2_ wagging, respectively [35]. The peak at 1316 cm^−1^ vanishes in doped samples, while the peak at 1410 cm^−1^ changes to 1422 cm^−1^ and 1468 cm^−1^ for samples including 15 and 30% (*v*/*v*), respectively. The O-H stretching vibration is responsible for a broad and intense absorption peak centered at 3339 cm^−1^ [36]. It is seen that the absorption peak at the 3339 cm^−1^ is a saturated flat pattern rather than a shoulder peak. This might be related to the thickness of the films as the FTIR is thickness dependent. The strong intra and inter type hydrogen bonding can be associated with the high intensity of this band [34]. This band shifts and its intensity are considerably reduced in doped materials. For the doped samples, the peak at 1644 cm^−1^, which is attributed to C=O stretching of the acetate groups, that is the remaining component of PVA, is changed to 1607 cm^−1^ [35]. At 2905 cm^−1^, the band analogous to C-H asymmetric stretching occurs [36]. For the loaded films, there is a noticeable change and substantial drop in this absorption band. The peak at 1076 cm^−1^ in Figure 4, which is a typical stretching vibration of –C–O– in pure PVA [37], is displaced to 1090 cm^−1^ and its strength diminishes.

### 3.3. XRD Analysis

**Figure 5:**
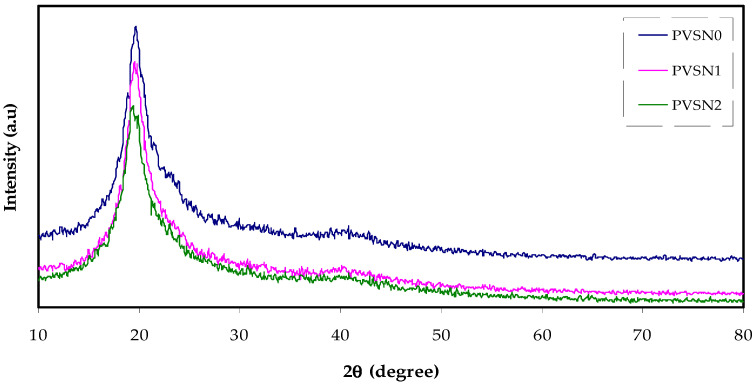
XRD spectra for pure PVA and doped films.

The XRD patterns of pure PVA and PVA doped with 30% (*v*/*v*) and 40% (*v*/*v*) of Sn^2+^-PPHs complex are shown in Figure 5. Pure PVA’s XRD pattern revealed a large peak about 20° that corresponded to the semi-crystalline structure of pure PVA [10]. A side from the major peak, two broad peaks may be found at 2θ = 23.4° and 41.18°. Based on the literature, the (101), (200) and (111) crystalline planes of PVA are responsible for the typical diffraction peaks at 2θ = 20°, 23.43° and 41.15°, respectively [37], and their shifts in the doped PVA sample are due to the complex formation between the functional groups of PVA and surface groups of the Sn^2+^-PPHs metal complex.

### 3.4. Absorption Study

The reaction of a substance to electromagnetic radiation, predominantly visible light, is referred to as an “optical property.” Sometimes it’s more practical to consider electromagnetic radiation (e.m.r) from the perspective of quantum physics, in which the e.m.r is considered as energy packets, i.e., as photons, instead of waves. The following relationship is used to quantify and characterize the energy *E* of a photon.

**Figure 6:**
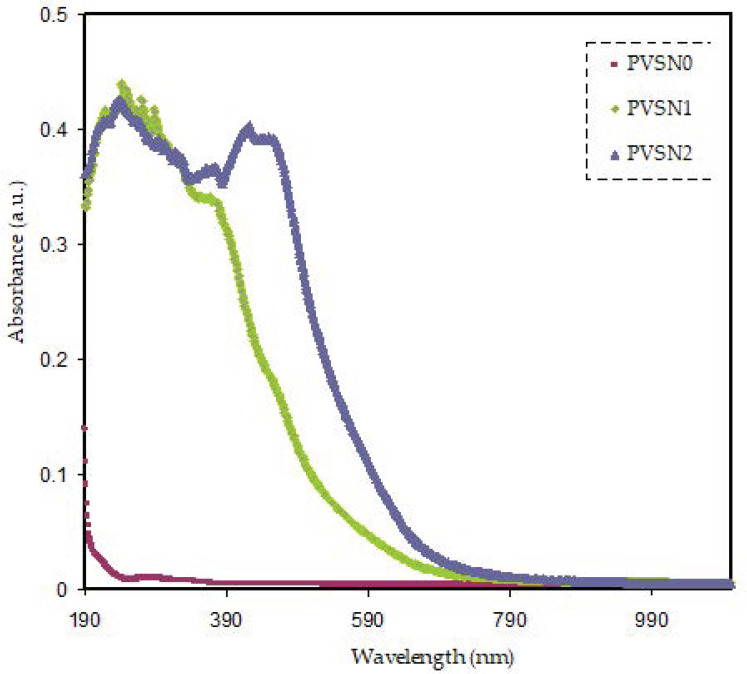
Absorption spectra for pure PVA and loaded films.

*E* = *hυ* = *hc/λ* (1)

where *h* stands for Planck constant (6.63 × 10^−34^ J/s), *c* is denotes to the light speed in free space(3 × 10^8^ m/s), and *λ* is the photon wavelength. Figure 6 illustrates the results. It is clear that the PCs absorption spectra include substantially all of the relevant areas of the UV-visible to NIR ranges. It is well known that the majority of the metal-complex compounds have excellent optical absorption and emission, with wavelengths extending from 600 to 700 nm [38]. This can be explained by the creation of orbital overlaps, which is aided by ligands (functional groups). As a result, electrons can transfer energy via the structure, which is what causes the absorption spectra [39]. The photon is not absorbed and the substance would be transparent to the photon when the incident photon energy is smaller than the energy difference between two levels of electrons. Absorption happens at higher photon energies (usually in 10^−15^ s) when the valence electrons transition between two electronic energy states [3].

The incorporation of metal-complexes into polymers for optoelectronic device and photonic device applications is said to be still under investigation [40]. Organic–inorganic composites have received a lot of attention as a potential material for a novel generation of nonlinear optical, electronic and optical instruments, along with biological labels [41].

### 3.5. Absorption Edge Study

The optical energy band gap (*E_g_*) of amorphous and crystalline materials can be estimated using the optical absorption spectra. The value and nature of the *E_g_* can be measured using fundamental absorption, which relates to electron excitation from valance to conduction band [42]. It is true that a light wave experiences losses or attenuation when it travels through a substance. The absorption coefficient, often known as the fractional reduction in intensity over distance, is calculated as follows [43]:

α = −1/*I* × d*I*/dx = (2.303/d) × *A* (2)

where, *I* denotes to the intensity and *A* is absorption quantity. The ultraviolet-visible (UV-vis) is valuable method for studying electronic transitions.

**Figure 7:**
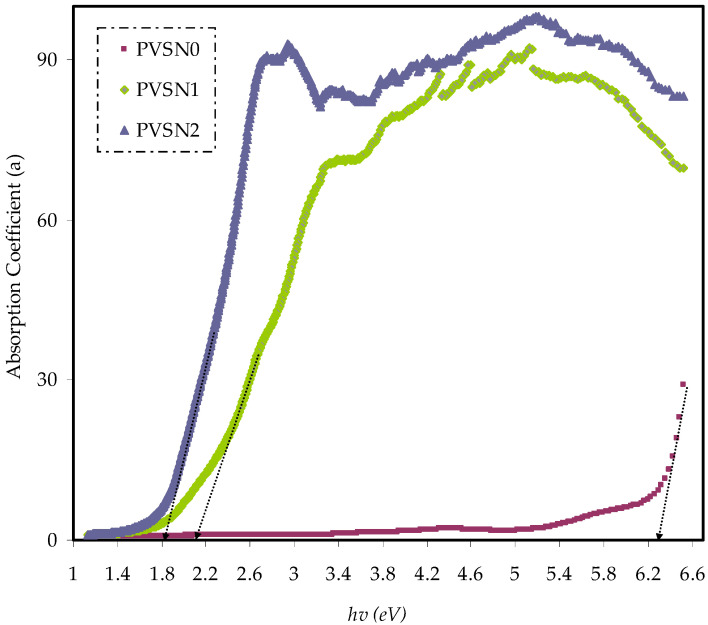
Absorption coefficient vs. *hυ* for PVA and loaded films.

When optical transitions begin to occur over a material’s fundamental band gap, the absorption edge is formed [44]. When PVA is transformed to tapered band gap polymer hybrid by integrating green produced metal-complex, a new study domain in optical materials is generated. Following that, a sole approach for polymer hybrid production using green technologies is developed. Figure 7 depicts the large absorption edge shift to lower photon energy. For the sample loaded with 30% (*v*/*v*) of Sn^2+^-PPHs metal complex, the value of absorption edge decreased substantially from 6.3 eV to 1.8 eV. (Figure 7). The absorption edge values are shown in Table 1. The absorption coefficient is determined using Equation (2). The intercept of the linear parts of the spectra of absorption coefficient with the axis of photon energy gives the value of the absorption edge. The absorption coefficient values reported in this work are very similar to those obtained for loaded polyacetylene (trans-(CH)_x_) and polypyrrole [45]. This is connected to the charge transfer complex creation in PC samples. Materials science has found molecule charge transfer materials to be an interesting and a good candidate for assessing molecular CT mechanisms, as well as changes in transport, magnetic, optical, dielectric, and structural properties. CT complexes have fascinating electrical, optical, and photoelectrical properties, and they have a good role in a variety of electro-physical and optical processes [46]. PMMA was doped with Alq3 by Duvenhageetet al. for application in optoelectronics [47]. There was a decrease in device performance and efficiency due to the quick breakdown of organometallic and conjugated polymers [47]. The color of the hybrid samples necessitates a change in the hybrid films’ band structure. Because there is enough evidence for a link between color and electrical structure in conductive polymers, polypyrrole’s small *E*_g_ can be predicted from its blackish color [45]. As a result, the optical property of polymeric materials is deduced from their color.

### 3.6. Refractive Index Study

**Figure 8:**
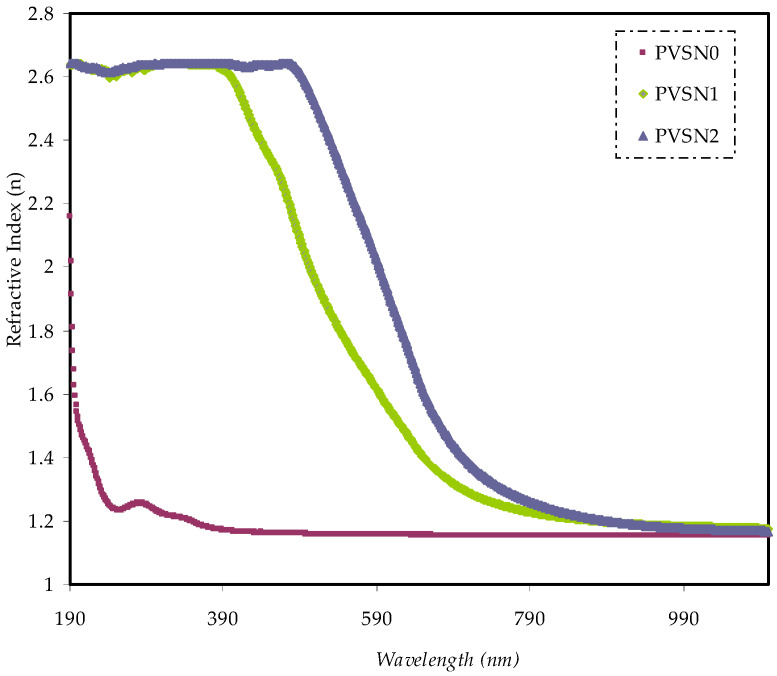
Refractive index spectra versus wavelength for PVA and loaded films.

The refractive index (*n*) and its dispersion behavior are two of the most essential features of an optical material. In optical communication and spectrum dispersion device design, refractive index dispersion is a vital element [48]. Some models are used to measure the optical *E_g_* using the dispersion area of *n*, as shown in the next section. Figure 8 depicts the value of (*n*) in relation to wavelength. It has been verified that higher *n* values are associated with integrated films that show significant dopant dispersion. It is seen that, as the % (*v*/*v*) of the Sn^2+^-PPHs metal complex rises, the value of *n* rises with it. The *n* is a function of both polarizability and density of a medium at constant temperature and pressure [49]. As a result, a material’s refractive index is one of the most important factors in measuring its optical efficiency. The following is an illustration of a samples complex refractive index:

(3) $$n^{*}\left(\lambda \right)=n\left(\lambda \right)+k\left(\lambda \right)$$

The *k* and *n* relationship is formulated as follow [35]:

(4) $$n=\left[\frac{\left(1+R\right)}{\left(1-R\right)}\right]+\sqrt{\frac{4\times R}{{\left(1-R\right)}^{2}}}-K^{2}$$

*K* is the extinction coefficient and is equal to *αλ*/4*πt* in Equations (3) and (4), where *t* is the sample thickness.

As photons are decelerated as they pass into a material because of interaction with electrons, the *n* is greater than one. The greater the *n* of a material, the more photons are retarded while passing through it. In general, any method that enhances a material’s electron density also enhances its refractive index [50]. Moreover, when compared to pure PVA, the dispersion behavior of refractive index verses wavelength can be seen for all doped films. This is the result of the doped samples’ density growing. Two methodologies are considered to improve the *n* value of polymers, depending on the method of synthesis: heavy atoms, for instance, polymers loaded with halogens and/or sulfur atoms [51], and the integration of metal or inorganic NPs into polymers to produce compounds with relatively high *n* values [52]. In all circumstances, there are two primary obstacles when manipulating *n*. To begin, the first way faces two challenges: the technological and financial difficulties of incorporating heavy atoms into polymer matrices [53]. Second, when inorganic NPs (ZrO_2_, TiO_2_ or Au NPs) are combined with nanofillers, aggregation occurs [52,54]. As a result, significant surface energy is formed, along with low compatibility with the polymer. In this work, Sn^2+^-PPHs metal complex was injected into the PVA polymer in order to modify the (*n*) value.

The single oscillator model proposed by Wemple and DiDomenico [55] is used to study refractive index dispersion (*n_o_*) in the normal dispersion zone. A dispersion energy parameter (*E_d_*) was incorporated into this model to represent the *n_o_*. It is a measure of the strength of the inter band optical transition. This parameter is directly related to chemical bonding and connects the charge distribution and coordination number through each unit cell [56]. Therefore, the energy of an oscillator is proportional to a single oscillator parameter (*E_o_*). This semi-empirical formula can be used to connect the refractive index to the photon energy below the interband absorption edge.

(5) $$n^{2}-1=\frac{E_{d}E_{o}}{\left[E_{o}{}^{2}-{\left(h\upsilon \right)}^{2}\right]}$$

**Figure 9:**
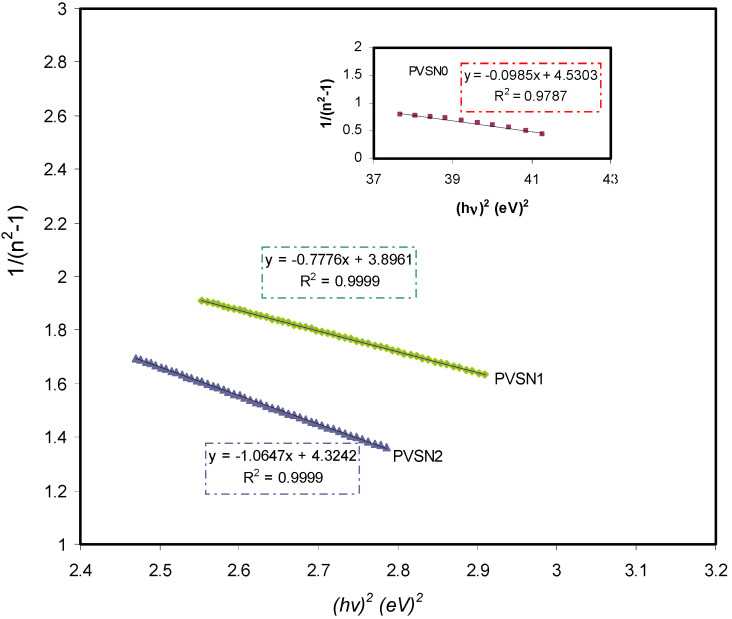
1/(n^2^ − 1)against (*hυ*)^2^ for PVA and loaded films.

Plotting **1/(*n*^2^ − 1)** against (*hυ*)^2^ yields the values of (*E_d_*) and (*E_o_*) from the slope and intercept of the linear fitted lines, as shown in Figure 9. Table 2 shows the *E_o_* and *E_d_* values that were calculated. The single oscillator energy (*E_o_*) declines as the % (*v*/*v*) of Sn^2+^-PPHs metal complex increases, whereas the dispersion energy (*E_d_*) rises. The static refractive index at zero energy *n_o_* is measured from the linear part extrapolation of Figure 9 to intersect the ordinates or is measured by $n_{0}=\sqrt{1+\frac{E_{d}}{E_{0}}}$. The oscillator energy *E_o_* is a “average” energy gap that, to a reasonable degree, is experimentally related with the lowest direct band gap [57]. As shown in Table 2, the overall image gained is consistent with the fact that refractive index and energy gap are inversely proportional.

### 3.7. Complex Dielectric Function Study

PC materials are one of the most reliable ways for modifying the dielectric constant (*ε_r_*) value of polymers. Several methods are now being used to improve the *ε_r_* of polymers, which can then be used in photonic or optoelectronic device applications. As demonstrated below, the *ε_r_* is approved in a connection that includes both values of (*k*) and (*n*) [58]:

(6) $$\varepsilon_{r}=n^{2}-k^{2}$$

**Figure 10:**
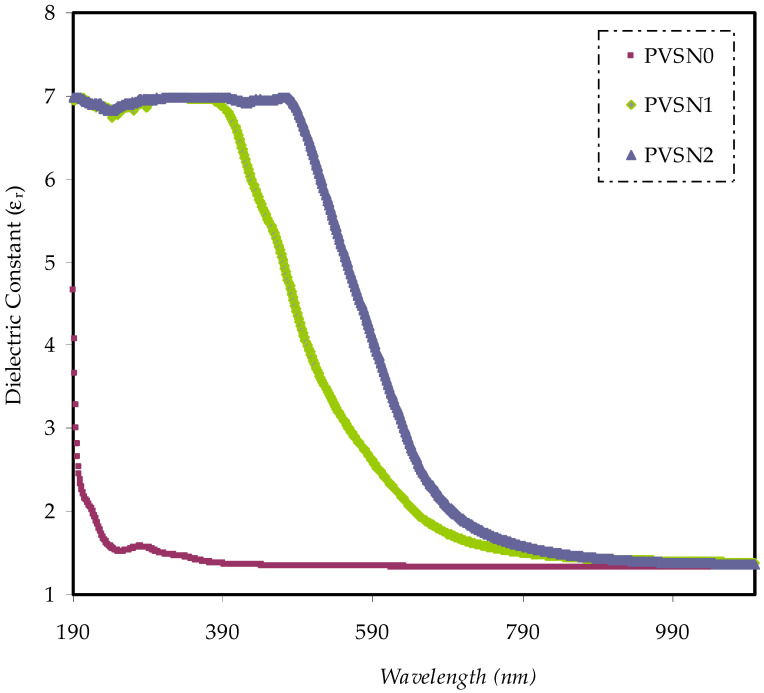
Dielectric constant versus wavelength for PVA and loaded films.

Figure 10 shows the *ε_r_* spectra against wavelength for PVA and PC samples. It is clear that, when the % (*v*/*v*) of Sn^2+^-PPHs metal complexes rises, the values of *ε_r_* rise as well. This is associated with the production of density of states inside the polymers, which is forbidden gap [58].

It is seen that the fundamental optical transition in PCs is caused by changes in the *ε_r_*. The response of this feature is reflected in the real (*ԑ_r_*) and imaginary (*ԑ_i_*) regions of the spectra. Contrastingly, the actual part determines a material’s ability to reduce the speed of an e.m.r wave. The imaginary part, on the other hand, indicates the level of energy absorption efficiency by materials as a result of polarization.

*Ε_r_* is wavelength dependent. As seen in Figure 10, dielectric constant is high at the low wavelength, while it has a low value at the long wavelength as more photons are absorbed at the low wavelength. Conducting polymers are expensive in comparison with the insulating polymers. In this research, Sn^2+^-complexes were added to the PVA polymer to increase the dielectric constant, as the Sn^2+^-complex has more functional groups to interact with the PVA polymer for increasing the dielectric constant and decreasing the *E_g_*. In addition to that Sn^2+^-complexes create trap energy states within the band gap that cause an increase in the value of the dielectric constant.

The *n* and wavelength connection, which is on the basis of the Spitzer–Fan model, can be used to specify the dielectric response (*ԑ_∞_*) of a substance at high frequency (i.e., short wavelength) [59]:

(7) $$\varepsilon_{r}=n^{2}-k^{2}=\varepsilon_{\infty }-(\frac{e^{2}}{4\pi^{2}C^{2}\varepsilon_{o}})\times (\frac{N}{m^{*}})\lambda^{2}$$

where *ԑ_o_* means the free space dielectric constant, *N* denotes the number of charge carrier, m* signifies the effective mass, which is presumed to be 1.16 m_e_, and *c* and *e* have their normal definitions [60].

**Figure 11:**
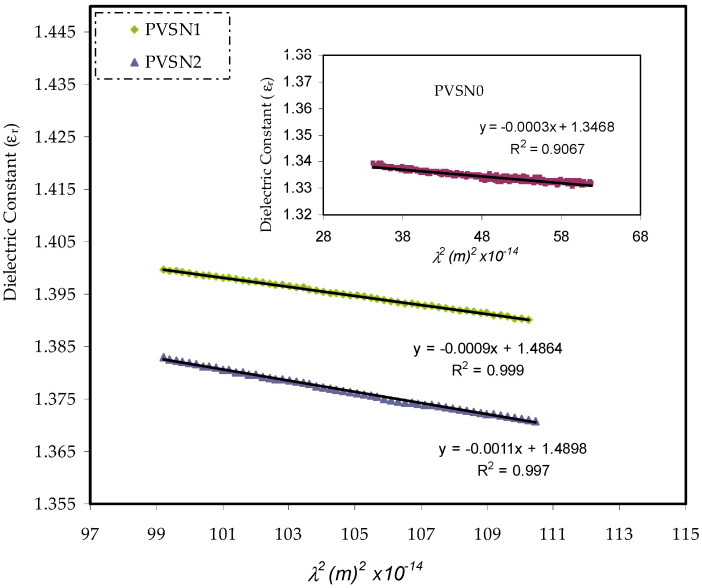
Shows the relationship of ԑ_r_ versus λ^2^ for pure PVA and doped films.

In the visible wavelength area, the relationship between the values of ԑ_r_ against λ^2^ is a straight line, as seen in Figure 11. Using the parameters in Table 3, one may calculate the *ԑ_∞_* and N/m* from the intercept and slope of the line with the vertical axis, correspondingly. Equation (7) can be used to approximate the N/m*, ԑ_∞_ and N, as shown in Table 4.

Table 4 shows that as the volume of the metal complexes increases, the charge carriers/m* of the parent PVA film increases, from 3.65 × 10^55^ to 13.38 × 10^55^ m^−3^ Kg^−1^ and the *ԑ_∞_* increases from 1.346 to 1.489. These increases in charge carriers/m^*^ and the *ԑ_∞_* is interpreted as indicative of a rise in the number of free charge carrier involved in the polarization mechanism. The calculated N/m* in this research are in good agreement with those documented in the previous reports by Equation (7) [61].

### 3.8. Band Gap Study

The clarification of atomic spectra particularly that of the simplest atom, hydrogen, was the first significant achievement of quantum theory. Quantum physics offered a vital concept: atoms could only immerse well-defined energy levels, and these energy states were exceedingly sharp for solitary atoms. Atoms cannot be seen as separate units in a crystalline solid because they are chemically connected to their nearest neighbor since they are in close proximity to one another. The nature of the chemical bond indicates that electrons on close adjacent atoms can exchange with one another, creating the spreading of discrete atomic energy states into energy ‘bands’ in the solid [62]. When considering a solid, it must take into account the contributions of numerous electronic energy band processes to the optical characteristics. Intraband (IBD) processes, for example, correspond to electronic conduction by free charge carriers and are more relevant in conducting materials such as semimetals, metals, and degenerate semiconductors. The classical Drude theory, or the Boltzmann equation, or the quantum mechanical density matrix method, can explain these IBD phenomena in their most basic terms [63]. Solid-state materials’ optical properties are useful for analyzing magnetic excitations, lattice vibrations, energy band structure, localized defects, impurity levels, and excitons. An electron is excited from a full valence band state to an empty conduction band state by a photon. An IBD transition is a quantum mechanical phenomenon [63]. Because of their scientific value and prospective application in energy conversion and harvesting, essential understanding of the charge separation and transfer procedures elaborate in photovoltaic systems is an exciting study topic that is gathering more and more attention [64]. The optical *E_g_* is the most essential property of organic and inorganic materials (*E_g_*)

**Figure 12:**
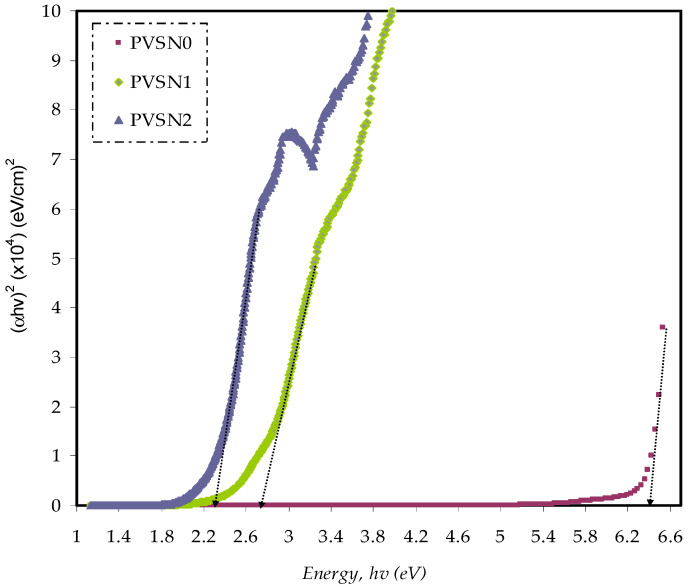
Plot of (*αhυ*)^2^ vs. *hυ* for pure PVA and PC films.

**Figure 13:**
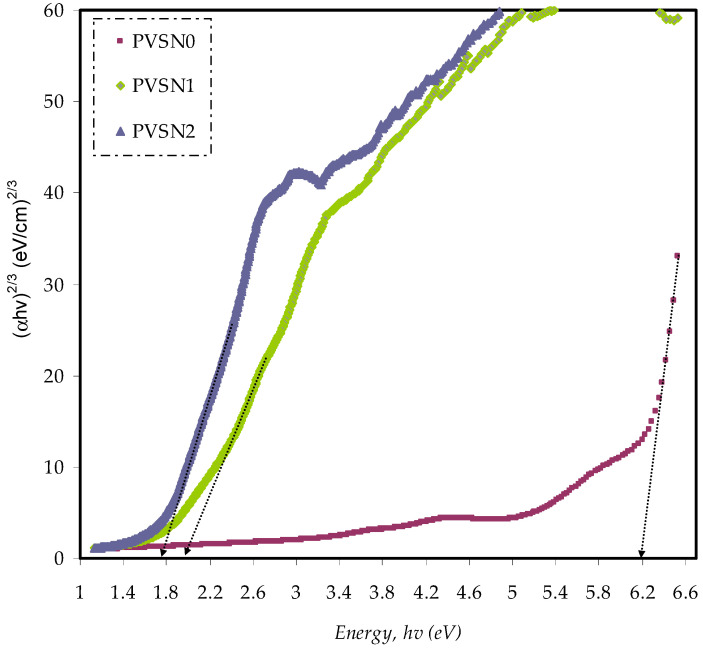
Plot of (*αhυ*)^2/3^ vs. *hυ* for pure PVA and PC films.

**Figure 14:**
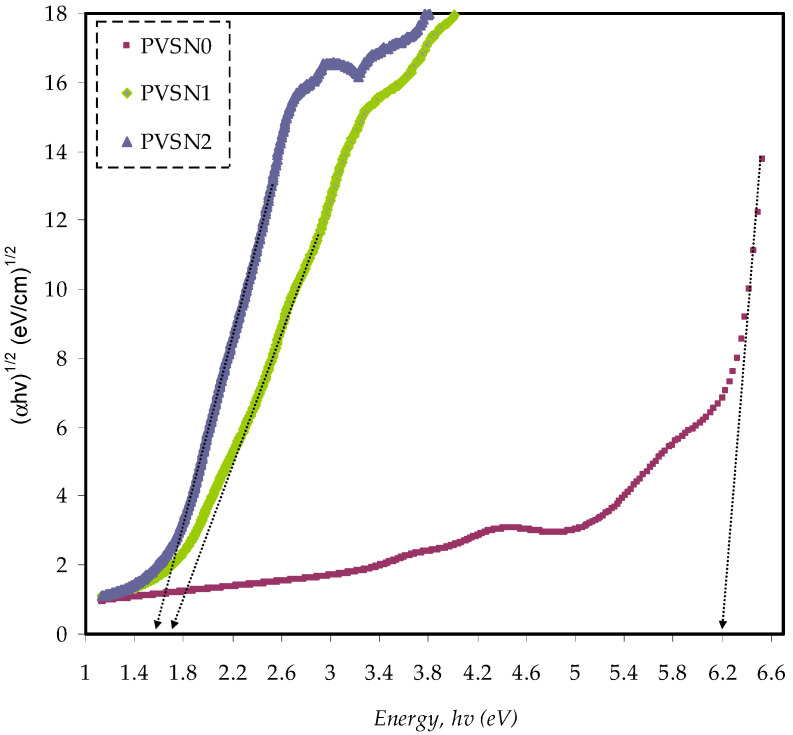
Plot of (*αhυ*)^1/2^ vs. *hυ* for pure PVA and PC films.

**Figure 15:**
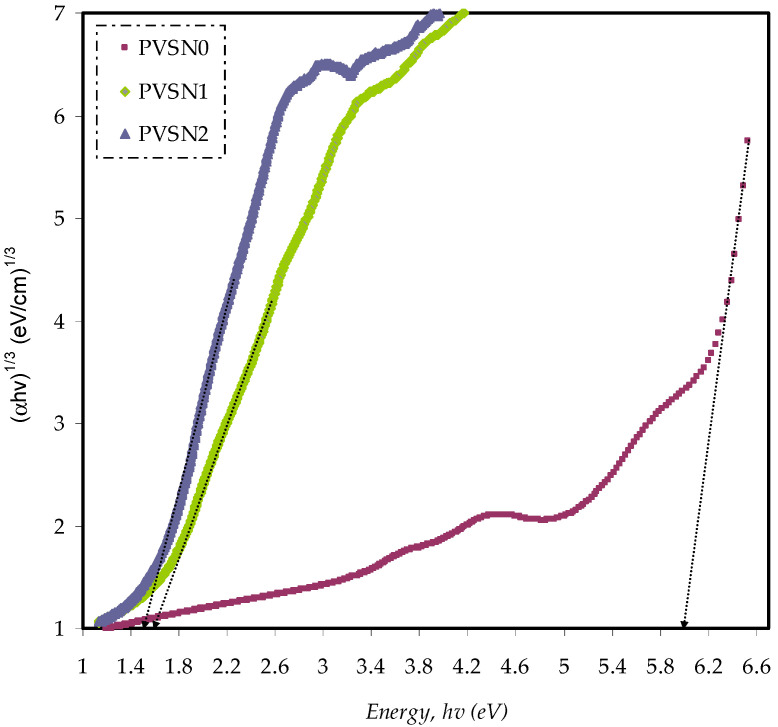
Plot of (*αhυ*)^1/3^ vs. *hυ* for pure PVA and PC films.

Tauc’s model [65] was used to calculate the energy band gap of the films.

(8) $$(\alpha h\upsilon )=B{\left(h\upsilon -E_{g}\right)}^{\gamma }$$

where *B* is a transition probability factor that is constant through the visible frequency ranges, and the index is utilized to measure the kind of electronic transition and takes 1/2 or 3/2 for direct transitions, while it is equal to 2 or 3 for indirect transitions, based on whether they are permitted or prohibited [66]. The plot of (*αhυ*)^1/γ^ against (*hυ*) for pure PVA and doped films is shown in Figure 12, Figure 13, Figure 14 and Figure 15.

When the Sn^2+^-PPHs was added to the PVA polymer, the optical energy bandgap decreased noticeably, as the Sn^2+^-PPHs are enriched with more functional groups to interact with the functional groups of PVA. Thus, the optical energy bandgap is noticeably decreased. For example, when 15 wt.% Sn^2+^-PPHs metal complex was added to pure PVA, the BGP reduced. For 30% (*v*/*v*) of inserted Sn^2+^-PPHs metal complex, a considerable modification in the energy band gap may be attained, lowering the BGP of PVA solid films to 1.8 eV. From the interception of the extrapolated linear component of the (*αhυ*)^1/^^γ^ on the photon energy axis, the optical energy band gap for all solid films was obtained (abscissa). Table 5 lists the optical BGP values. In insulator materials, the *E_g_* is too big that no free carriers can thermally excite over it at room temperature. This means there is not any carrier absorption. IBD transitions seem to be essential only at rather high photon energy, as a result (above the visible). Many insulator materials are optically transparent as a result of this. The findings show that PCs with low bandgap energies (1–2 eV) may be made, which has piqued scientists’ attention because to their potential applications in visible and infrared detectors, optical parametric oscillators, up converters, and solar cells [67].

The complex dielectric function, which is connected with other optical characteristics (i.e., *n*, reflectivity, and absorption coefficient) by simple equations, is the best way to characterize the optical properties of solids [68]. Electronic transition and charge transport complexes in semiconducting/conducting polymers are not studied well. The transitions are made possible by the incident photon and phonon giving sufficient energy and momentum [69]. Tauc’s model and optical dielectric loss have already been shown to be active in determining the *E_g_* and electronic transition types, respectively. This is due to the optical dielectric function is mostly independent of the materials band structure. Simultaneously, study of optical dielectric function utilizing UV–vis spectroscopy have proven to be relatively valued in foreseeing the materials band structure [10,25]. Aside from IBD (free carrier) activities, interband processes occur when electrons in a filled level below the Fermi state absorbs electromagnetic radiation, causing a transition to the unfilled level in a higher band. This IBD process is fundamentally a quantum mechanics method that is explained using quantum mechanical terminology [63].

The *ԑ_i_* can be determined experimentally from the given *n* and *k* data using the following relationships,

ԑ_2_ = 2 *n k* (9)

**Figure 16:**
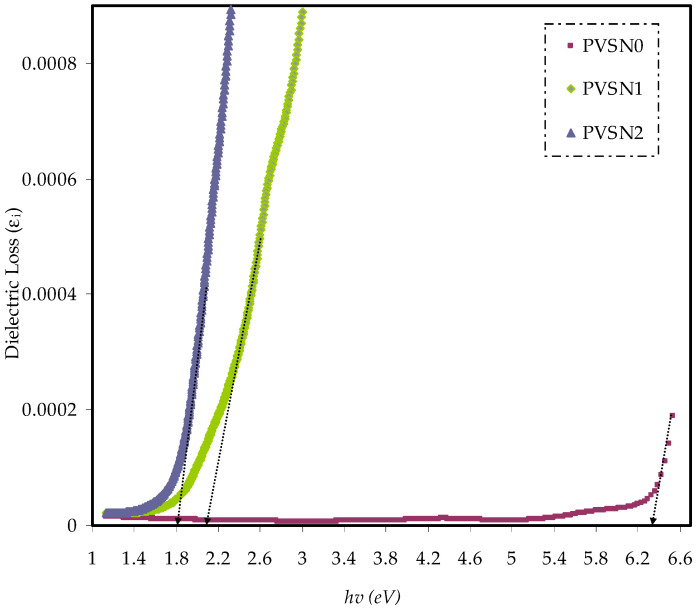
Plot of *ԑ_i_* vs. *hυ* for PVA and PC films.

The refractive index is *n*, while the extinction coefficient is *k*. Previous research has shown that the peaks in the *ԑ_i_* spectra are linked to the interband transitions [34,35,36]. The real *E_g_* can thus be calculated by taking the intersection of linear sections of *ԑ_i_* spectrawith the *hυ* axis (see Figure 16). This is because the optical dielectric function is intimately linked to the photon–electron interaction and relates the physical process of IBD transition through the structure of electronic materials. The dielectric function’s imaginary part(*ԑ_i_*) primarily describes the electron transition from filled to unfilled levels [70]. Former work documented that studying the *ԑ_i_* allowed for a detailed understanding of the optical transition mechanism [71]. An electron is excited by a photon from a valence band occupied state to a conduction band unoccupied state. This is referred as IBD transitions. A photon is absorbed in this method, which results in the formation of a hole and an excited electronic level. Quantum mechanics governs this process [63]. The optical dielectric loss is significantly connected to the filled and unfilled electronic levels within a solid from a quantum mechanics (microscopic) standpoint. The peak in the imaginary component of the dielectric function correlates to strong IBD transitions, which is well documented microscopically (quantum mechanically) [70]. The study of the complex dielectric function (ԑ* = *ԑ_r_* − *iԑ_i_*), which defines the material linear response to e.m.r, will help you better comprehend the optical properties of a solid. The imaginary component *ԑ_i_* represents the material’s optical absorption, which is tightly linked to the valence (filled) and conduction (unfilled) bands, and is given by [18]:

(10) $$\varepsilon_{2}(\omega )=\frac{2e^{2}\pi }{\Omega \varepsilon_{o}}\sum_{K,V,C}\left|\psi_{K}^{C}\right|\vec{U}\cdot \vec{r}|{\psi_{K}^{V}|}^{2}\delta \left(E_{K}^{C}-E_{K}^{V}-\hbar \omega \right)$$

where *ω*, Ω, *e*, *ԑ_o_*, $\vec{r}$ and $\vec{u}$ are the incident photon frequency, crystal volume, electron charge, free space permittivity, position vector, and a vector determined by the incident e.m.r. wave polarization, respectively. $\psi_{k}^{c}$ and $\psi_{k}^{v}$ are the conduction band wave function and valence band wave function, respectively, at k. The optical dielectric constant is characterized by a complex function of frequency based on theoretical models, which necessitates a large-scale computer effort to calculate [71,72]. By comparing the *ԑ*” of Figure 16 to those extracted from Tauc’s method, the types of electronic transition is recognized (Figure 12, Figure 13, Figure 14 and Figure 15). It is feasible to determine that the kind of electronic transition in pure PVA and PC samples is direct allowed (γ = 1/2) and direct forbidden (γ = 3/2) transitions, respectively. Table 5 summarizes the band gap calculated using optical dielectric loss and the Tauc technique for more clarity.

## 4. Conclusions

In conclusion, the PVA films doped with Sn^2+^-PPHs metal complex were synthesized with low optical energy band gaps by solution casting procedure. The FTIR showed that the BT contained sufficient PPHs and functional groups to fabricate Sn^2+^-PPHs metal complex. The UV-vis and FTIR methods confirmed the formation of Sn^2+^-PPHs metal complex. The UV-visible method showed the effect of the Sn^2+^-PPHs metal complex on the optical property of PVA. Furthermore, XRD and FTIR analyses showed the formation of complexation between PVA and Sn^2+^-PPHs metal complex. The improvement of the amorphous structure is reflected in the broadness and decrease in the XRD intensity. The shifts and decreases in the intensity of the FTIR peaks of the composite films established the interaction between Sn^2+^-PPHs metal complex and PVA. The absorption edge shifted to lower *hυ* by increasing the load of the Sn^2+^-PPHs metal complex. The refractive index and dielectric constant tuned by loading of Sn^2+^-PPHs metal complex to PVA. The *E_o_*, *E_d_* and *n_o_* were calculated for the films. The dielectric constant versus photon wavelength were studied to measure *N*/m* and high frequency dielectric constant. Tauc’s model was used to measure the type of electronic transition in the films. To estimate the energy gap, the dielectric loss parameter was analyzed. Because of the low optical band gap of the films, these films have good potential for optoelectronic device applications.

## Figures and Tables

**Table 1:** Absorption edge for PVA and loaded films.

Sample Code	Absorption Edge (eV)
PVSN0	6.3
PVSN1	2.1
PVSN2	1.8

**Table 2:** *E_o_* and *E_d_* for the PVA and loaded films.

Sample	*E_d_*	*E_o_*	*n_o_*
PVSN0	1.49	6.74	1.221
PVSN1	0.57	2.22	1.256
PVSN2	0.46	1.98	1.232

**Table 3:** The physical quantities used to determine N/m* for PVA loaded Sn^2+^ metal complex.

Physical Parameters	Values
*m_e_*	9.109 × 10^−31^ Kg
*E*	1.602 × 10^−19^ coulombs
*ԑ_o_*	8.85 × 10^−12^ F/m
*π*	3.14
*C*	2.99 × 10^8^ m/s
m*	10.566 × 10^−31^ Kg

**Table 4:** Presents the values of N/m* and *ԑ_∞_* for PVA loadedSn^2+^-complex.

Film Code	N/m* × 10^55^ (m^−3^/kg)	*ԑ_∞_*
PVSN0	3.65	1.346
PVSN1	10.94	1.486
PVSN2	13.38	1.489

**Table 5:** Opticalbandgap from Tauc’smodel and dielectric loss plot.

Sample Code	γ = 1/2	γ = 3/2	γ = 2	γ = 3	*E_g_* From *ԑ_i_*
PVSN0	6.4	6.19	6.08	6	6.4
PVSN1	2.74	2	1.82	1.6	2.1
PVSN2	2.3	1.78	1.6	1.56	1.8

## Data Availability

Not applicable.
